# Disorders of Human Coenzyme Q10 Metabolism: An Overview

**DOI:** 10.3390/ijms21186695

**Published:** 2020-09-13

**Authors:** Iain Hargreaves, Robert A. Heaton, David Mantle

**Affiliations:** 1 School of Pharmacy, Liverpool John Moores University, L3 5UA Liverpool, UK; R.Heaton@2013.ljmu.ac.uk; 2 Pharma Nord (UK) Ltd., Telford Court, Morpeth, NE61 2DB Northumberland, UK; dmantle@pharmanord.com

**Keywords:** coenzyme Q10, mitochondria, oxidative stress, antioxidant, deficiencies

## Abstract

Coenzyme Q10 (CoQ10) has a number of vital functions in all cells, both mitochondrial and extramitochondrial. In addition to its key role in mitochondrial oxidative phosphorylation, CoQ10 serves as a lipid soluble antioxidant, plays an important role in fatty acid, pyrimidine and lysosomal metabolism, as well as directly mediating the expression of a number of genes, including those involved in inflammation. In view of the central role of CoQ10 in cellular metabolism, it is unsurprising that a CoQ10 deficiency is linked to the pathogenesis of a range of disorders. CoQ10 deficiency is broadly classified into primary or secondary deficiencies. Primary deficiencies result from genetic defects in the multi-step biochemical pathway of CoQ10 synthesis, whereas secondary deficiencies can occur as result of other diseases or certain pharmacotherapies. In this article we have reviewed the clinical consequences of primary and secondary CoQ10 deficiencies, as well as providing some examples of the successful use of CoQ10 supplementation in the treatment of disease.

## 1. Introduction

**Figure 1:**
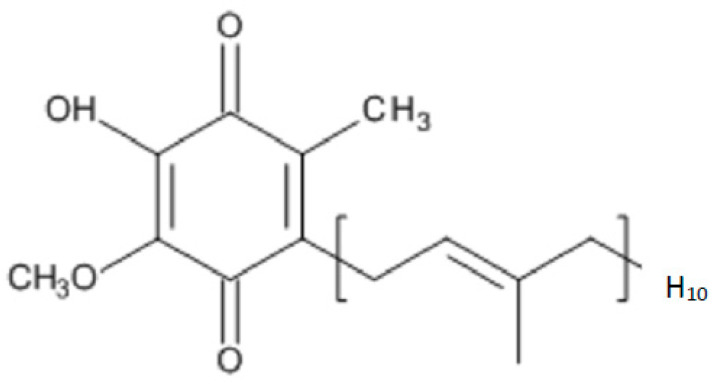
The chemical structure of CoQ_10_.

Coenzyme Q10 (CoQ10) is a lipid-soluble molecule comprising a central benzoquinone moiety, to which is attached a 10-unit polyisoprenoid lipid tail [1] (Figure 1). The benzoquinone ring contains redox active sites, whereas the polyisoprenoid chain is responsible for positioning the CoQ10 molecule within the mid-plane of the lipid bilayer of various cell membrane types. CoQ10 is usually described as a vitamin-like substance, although by definition, CoQ10 is not a vitamin, since it is produced by various tissues within the human body. The synthesis of the CoQ10 molecule comprises three major steps, namely, synthesis of the benzoquinone structure from 4-hydroxybenzoate (derived from either tyrosine or phenylalanine), synthesis of the polyisoprenoid side chain from acetyl-coenzyme A (CoA) via the mevalonate pathway, and condensation of these two structures to form coenzyme Q10; the benzoquinone ring structure is then subject to further modification via hydroxylation, methylation and decarboxylation to form CoQ10 [2]. There are several potential rate-limiting steps in the overall CoQ10 biosynthetic pathway, including synthesis of the polyisoprenoid chain (via HMG-CoA reductase) and condensation of the polyisoprenoid chain and benzoquinone ring (via prenyltransferase) [3].

**Figure 2:**
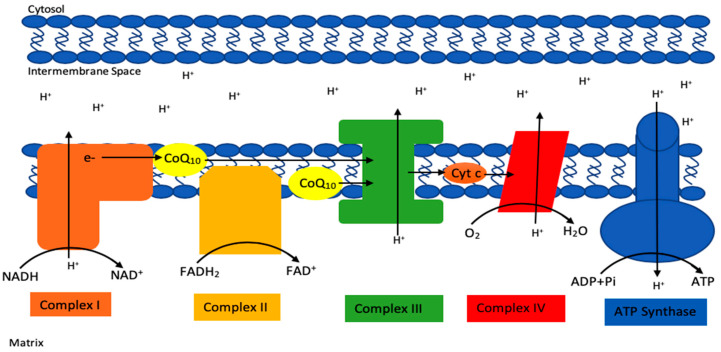
Basic schematic of the mitochondrial electron transport chain (METC), highlighting the electron carrier function of CoQ10 in the chain.

CoQ10 has a number of vital cellular functions, particularly within mitochondria, but also elsewhere within the cell [1]. Within mitochondria, CoQ10 has a key role as an electron carrier (from complex I and II to complex III) in the mitochondrial electron transport chain (METC) during oxidative phosphorylation (Figure 2). It is also involved (as a cofactor of the enzyme dihydroorate dehydrogenase) in the metabolism of pyrimidines, fatty acids and mitochondrial uncoupling proteins, as well as in the regulation of the mitochondrial permeability transition pore [1]. CoQ10 serves as an important lipid-soluble antioxidant protecting cellular membranes, both mitochondrial and extra-mitochondrial (Golgi apparatus, lysosomes, endoplasmic reticulum, peroxisomes) from free radical-induced oxidative stress (OS) [2]. In addition to acting as an antioxidant directly, CoQ10 is also involved in the regeneration of the antioxidants vitamin C and vitamin E, respectively [4]. In addition, CoQ10 has a role as a mediator of inflammation, a role in cholesterol metabolism [5], a role in maintaining lysosomal pH [6], a role in sulphide metabolism as a cofactor of the sulphide quinone oxidoreductase [7] and a role in amino acid metabolism (as a co-factor of choline dehydrogenase and proline dehydrogenase in the synthesis of glycine and proline/arginine, respectively) [8,9]. CoQ10 has been shown to directly affect the expression of a number of genes [10]. CoQ10 exists in both oxidised (ubiquinone) and reduced (ubiquinol) forms, and the normal functioning of CoQ10 involves continual inter-conversion between these two forms [1].

The daily requirement for CoQ10 is not known with certainty but has been estimated to be approximately 500 mg/day, based on a total body pool of 2000 mg and average tissue turnover time of four days [11]. A small amount of CoQ10 (approximately 5 mg) is obtained from the daily diet [11], with most of the daily requirement being synthesised within the body. CoQ10 is produced in many tissues, being at particularly high levels in the kidney, heart, skeletal muscle and liver [12]. However, when taking into account tissue CoQ10 levels (μg/g of tissue) together with organ weight, the liver appears to be the principal site of CoQ10 biosynthesis in the body. Optimal production occurs around 25 years of age, after which production steadily declines, with the production level at age 65 being approximately 50% of that at age 25 [13].

In addition to the effect of ageing, CoQ10 levels are also reduced by certain prescribed drugs (particularly statins), and in a variety of diseases [14]. Moreover, because CoQ10 has so many key roles in cell metabolism, a deficiency—whether caused by ageing, pharmaceutical drugs or illness—has profound effects on an individual’s health status. In this article, we have provided an overview of the causes and consequences of CoQ10 deficiency, and the role of CoQ10 supplementation in correcting such deficiencies.

## 2. Deficiency of CoQ10

### 2.1. Primary CoQ10 Deficiency

**Figure 3:**
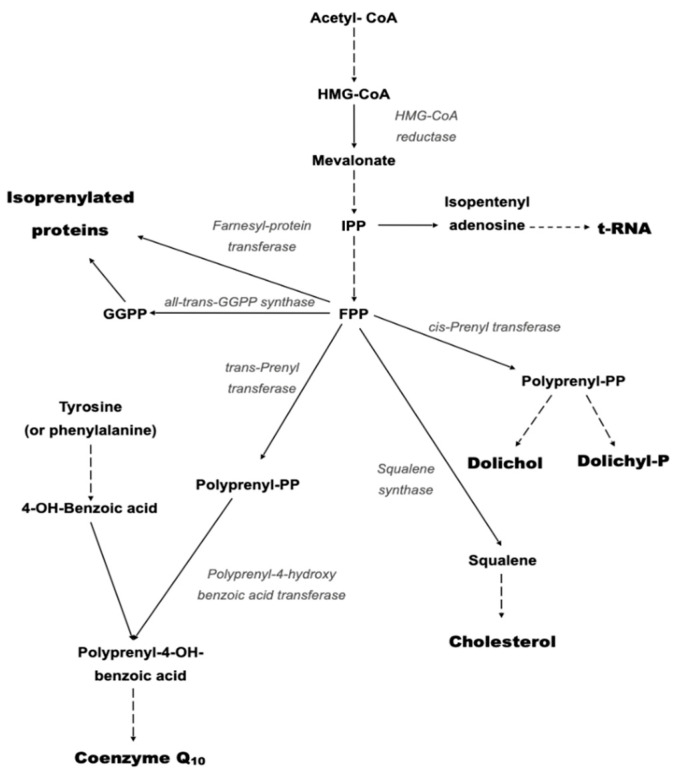
Schematic of the mevalonate pathway responsible for the biosynthesis of CoQ10. Solid arrows represent single biosynthetic reactions; Dashed arrows represent multiple biosynthetic reactions.

Deficiency of CoQ10 is broadly divided into primary CoQ10 deficiency and secondary CoQ10 deficiency [15]. Primary CoQ10 deficiency results from mutations in genes involved in the CoQ10 biosynthetic pathway [16]. The biosynthesis of CoQ is a complex multi-step process which takes place in various sub-cellular locations [17]. The polyisoprenoid tail is synthesised in the cytosol via the mevalonate pathway, with attachment to the benzoquinone ring (originating from tyrosine) taking place within mitochondria [17] (Figure 3).

At least 10 genes are required for the biosynthesis of functional CoQ10, a mutation in any one of which can result in a deficit in CoQ10 status [16]. The preponderance of the data relating to CoQ10 biosynthesis has been obtained from studies in yeast, with deficiencies corresponding to the above genes denoted as *CoQ1* to *CoQ11* (numbering refers to date order of identification) [18]. With regard to the corresponding enzymic/protein gene products, COQ1 (heterotetrameric decaprenyl diphosphate synthase, comprising PDSS1 and PDSS2) is involved in the synthesis of the polyisoprenoid chain, and COQ2 in the condensation of the isoprenoid chain with the benzoquinone ring [19]. COQ3, COQ5, COQ6 and COQ7 are involved in concomitant methylation, decarboxylation, hydroxylation and deamination reactions [20,21,22,23]. COQ8A is necessary for phosphorylation of COQs 3, 5 and 7 [24]. The COQ9 lipid-binding protein is necessary for stabilisation of COQ7 [25], and COQ10 directs the localisation of CoQ10 within the mitochondrial membrane [26].

In humans, mutations in 10 of these genes have been identified to date: the corresponding gene products respectively are PDSS1 (phenyl diphosdphate synthase subunit 1; [27]), PDSS2 (decaprenyl diphosphate synthase subunit 2; [28]) COQ4 (multienzyme complex organisation enzyme; [29]), COQ5 (methyltransferase; [30]), COQ6 (monooxygenase; [31]), COQ7 (DMG hydroxylase; [32], ADCK3 (renamed COQ8A, protein kinase; [33]), ADCK4 (renamed COQ8B, protein kinase; [34]) and COQ9 (lipid-binding protein; [35]).

### 2.2. Secondary CoQ10 Deficiency

Secondary coenzyme Q10 deficiency results from mutations in genes that are not directly related to the CoQ10 biosynthetic pathway, or to non-genetic factors associated with various disorders [36]. Examples of secondary CoQ10 deficiency resulting from such genes include mutations in the *APTX* gene encoding the protein aprataxin in ataxia oculomotor aprataxin 1 disorder [37], and the *BRAF* gene encoding the enzyme serine/threonine-protein kinase B-Raf in cardiofaciocutaneous syndrome [38], multiple acyl-CoA dehydrogenase deficiency (by mutations in the *ETFDH* gene; [39]) and spinocerebellar ataxia-10 (by mutations in the *AN010* gene; [40]). In addition, secondary CoQ10 deficiencies associated with a number of disorders have been reported, including primary METC disease and mitochondrial DNA depletion syndrome [41,42], cardiovascular disease [43], chronic kidney disease [44], type II diabetes [45] and metabolic syndrome [46]. Recently, evidence of a plasma CoQ10 deficiency has been reported in the lysosomal storage disorder mucopolysaccharidosis (MPS), as well the metabolic disease phenylketonuria (PKU) [47]. In addition, a deficiency in CoQ10 status is associated with aberrant sulphide metabolism [7]. Plasma CoQ10 status reflects both hepatic synthesis and dietary intake, and therefore it may not truly represent the cellular level of this isoprenoid [2,47]. However, in the case of MPS, evidence of a deficit in fibroblast CoQ10 status has been reported in patients with Sanfilippo A and B (MPS III), although there was no detectable impairment in the CoQ10 biosynthetic pathway [48]. In primary METC disorders, an aberrant ETC may affect the structural integrity or formation of the CoQ10 biosynthetic enzyme complex, which is located in the inner mitochondrial membrane close to the METC, and may therefore cause an impairment in the synthesis of this isoprenoid [49].

The factors responsible for inducing a deficit in CoQ10 status in secondary CoQ10 deficiencies are currently not completely understood, but in some cases may be disease-specific, for example the inhibition of the mevalonate pathway by high phenylalanine concentrations in PKU, or the low blood levels of vitamin B_6_ reported in MPS patients—the active form of vitamin B_6_, pyridoxal 5-phosphate, is an important cofactor required by the CoQ10 biosynthetic pathway [47].

**Figure 4:**
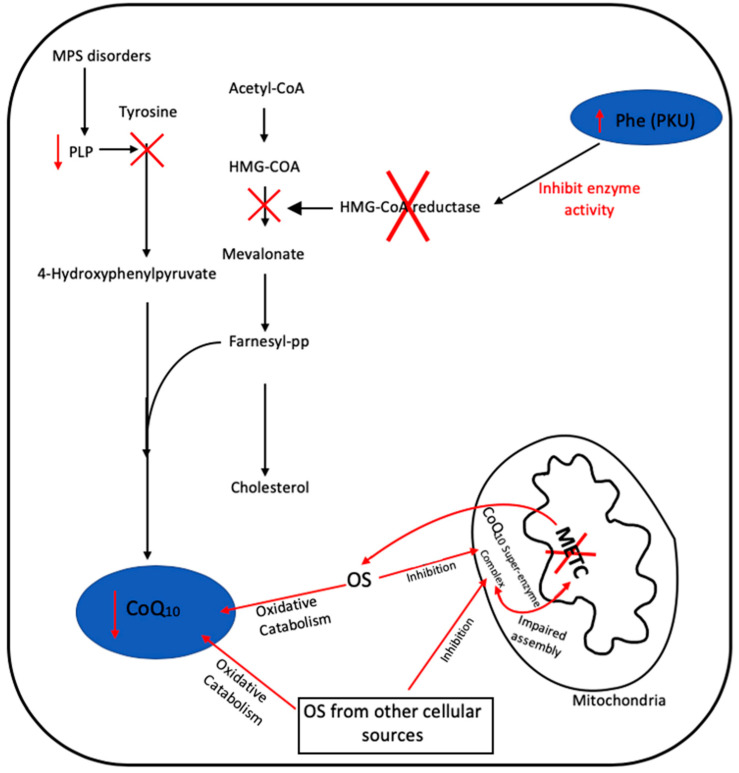
Putative mechanisms responsible for secondary CoQ_10_ in disease in MPS, PKU and METC disorders. Oxidative stress (OS), pyridoxal 5-phosphate (PLP), mucopolysaccharidosis (MPS), phenylketonuria (PKU), phenylalanine (Phe) and mitochondrial electron transport chain (METC). Black arrows represent defined biochemical reactions; red arrows represent tentative biochemical reactions. ‘X‘ represents the inhibition of an enzyme or biosynthetic step.

OS-induced degradation of CoQ10 has been suggested as a possible contributory factor to the deficit in the level of this isoprenoid reported in certain diseases, although this has yet to be confirmed or refuted [49]. In addition, the possibility arises that OS may also inhibit the enzymes of the CoQ10 biosynthetic pathway, which may also cause a diminution in cellular CoQ10 status, although this putative mechanism has yet to be investigated. Figure 4 outlines the possible causes of secondary CoQ10 deficiency in PKU, MPS and METC disorders.

A secondary deficiency in CoQ10 status has also been associated with certain pharmacotherapies such as statins, and the anti-depressant amitriptyline (AM) [50]. Statins target the liver and competitively inhibit the enzyme HMG-CoA reductase, the rate limiting enzyme in cholesterol synthesis [14]. In view of the commonality of the cholesterol and CoQ10 biosynthetic pathways, a deficiency in HMG-CoA reductase activity has the potential to impair CoQ10 biosynthesis (Figure 3).

The cause of CoQ10 deficiency associated with AM therapy is as yet unknown, although it may be caused by the increase in OS associated with this pharmacotherapy [50]. However, the loss of cellular mitochondrial enrichment associated with AM treatment may be an important factor to consider, since appropriately 50% of cellular CoQ10 resides within the mitochondria [2,51].

## 3. Clinical Consequences of a CoQ10 Deficiency

As noted from the known functions of CoQ10 summarised above, a deficiency in CoQ10 status can result in METC defects and impaired CoQ1 heterotetramer pdss1–pdss2 cellular ATP production, impaired antioxidant defense against free radical-induced oxidative stress, and impaired pyrimidine synthesis [41]. Reduced levels of endogenous CoQ10 in older individuals, with concomitant impairment of oxidative phosphorylation and antioxidant capacities, has been implicated in the age-related decline in normal tissue functions [43]. A CoQ10 deficiency may also cause a defect in the metabolism of hydrogen sulphide as the result of a reduction in the protein level of the enzyme quinone oxidoreductase, which is a sulphide, and a consequent alteration in mitochondrial sulphide metabolism [52,53].

Primary CoQ10 deficiency can affect any part of the body, but particularly the brain, muscle and kidney tissues, as a consequence of their high energy demands. The severity and time frame of symptoms are variable; severe symptoms may be evident in infancy, whereas mild symptoms may not become apparent until the individual is in their 60s [16]. CoQ10 deficiency in brain tissue can cause ataxia, together with a range of other neurological manifestations. CoQ10 deficiency in kidney tissue results in nephrotic syndrome and renal dysfunction, and deficiency in cardiac tissue results in weakened heart muscle, characteristic of hypertrophic cardiomyopathy [16].

The first cases of CoQ10 deficiency were reported by Ogasahara et al. [54]. The patients were two sisters born to unrelated parents, who presented with recurrent rhabdomyolysis associated with seizures and developmental delay. Since this report, a number of patients have been described, and although a CoQ10 deficiency appears to have a particularly heterogeneous clinical presentation, there appear to be five distinct clinical phenotypes: encephalomyopathy, severe infantile multisystemic disease, nephropathy, cerebellar ataxia and atrophy, and isolated myopathy [55]. Interestingly, cerebellar ataxia and atrophy has emerged as the most common clinical presentation of CoQ10 deficiency, and this may result from the low level of CoQ10 reported in the cerebellum compared to that of other brain regions [55,56].

Secondary deficiencies of CoQ10 typically occur in the mitochondrial myopathies [41,49], cardiovascular disease [57], type II diabetes [45], chronic kidney disease [44], liver disease [43] and critical illness [43]. Depletion of CoQ10 in these disorders may compromise cellular antioxidant status and result in impaired mitochondrial function and cellular energy supply, resulting in, for example, heart failure. However, the actual contribution of a deficit in CoQ10 status to the pathophysiology of these disorders has yet to be fully elucidated.

In statin therapy, a deficiency in CoQ10 status has been suggested as a possible contributing factor to the myopathic side effects associated with this pharmacotherapy [58]. Although a number of studies have reported evidence of a deficit in plasma/serum CoQ10 following statin therapy, few studies have directly assessed muscle status, and of these, only one has reported evidence of CoQ10 deficiency [14,59]. The decrease in circulatory CoQ10 status following statin therapy may reflect the decrease in blood low density lipoprotein (LDL) status induced by this pharmacotherapy [14]. LDL is the major carrier of CoQ10 in the circulation [2].

## 4. Clinical Assessment of CoQ10

The determination of CoQ10 is not usually included as part of the routine biochemical analysis of blood by hospital pathology laboratories. The most common analytical techniques used to assess CoQ10 status are based on high-pressure liquid chromatography (HPLC) with either ultraviolet (HPLC-UV) or electrochemical (HPLC-ED) detection [60]. CoQ10 levels are usually determined in plasma with reference levels typically in the range of 0.5–1.7 mM [61]. Moreover, in view of the fact that plasma CoQ10 status is dependent on the circulatory lipoprotein levels (as the major carrier of CoQ10 in the circulation). It has been suggested that in order to take into account the lipoprotein concentration of the blood, plasma CoQ10 levels should be expressed as a ratio to the total circulatory cholesterol status [2].

At present, there is uncertainty as to whether plasma CoQ10 status, which is the result of dietary intake and hepatic synthesis, actually reflects that of other tissues, and therefore whether it is an appropriate surrogate for use in clinical assessment [60]. Where tissue CoQ10 levels are to be determined, skeletal muscle is the usual tissue of choice, whereas skin fibroblasts have also been used [62]. CoQ10 levels in tissues and cells are typically expressed as pmol/mg (of protein; [60]). Typical reference ranges for skeletal muscle and fibroblasts CoQ10 status are 140–580 pmol/mg and 39–75 pmol/mg, respectively [63]. In addition to expressing tissue/cellular CoQ10 status as pmol/mg, some laboratories also present these values as a ratio to activity of the mitochondrial marker enzyme citrate synthase (CS) [60]. Since approximately 50% of cellular CoQ10 is present in mitochondria [64], expressing CoQ10 status to CS activity may be important to consider diagnostically. In mitochondrial myopathies, where the excessive proliferation of mitochondria has been reported, evidence of CoQ10 deficiency may only be identified when CoQ10 status is related to CS activity [60].

In view of the preponderance of neurological dysfunction associated with a CoQ10 deficiency, it would appear judicious to assess cerebral CoQ10 status in patients suspected to have this deficit. Cerebral spinal fluid (CSF) is considered the appropriate surrogate to evaluate cerebral CoQ10 status, and in view of the low levels of CoQ10 present in this matrix, only highly sensitive analytical techniques such as HPLC-ED and tandem mass spectrometry are appropriate for this analysis [60]. A tentative reference range of 5.7–9.0 nM has been established for CSF CoQ10 status [65]. Recently, a new methodology for the measurement of CoQ10 in urine has been standardised, including the establishment of reference values for a paediatric control population [66].

At present, there are studies in progress to develop an accurate method of determining blood spot (BS) CoQ10 status, enabling the assessment of this isoprenoid to be undertaken during new-born screening, therefore allowing a window of opportunity for therapeutic CoQ10 supplementation to be undertaken before irreversible organ dysfunction has occurred. However, until a reliable BS CoQ10 detection method has been developed, it is recommended that CoQ10 status is determined at the earliest opportunity in suspected patients to optimise therapeutic intervention [60]. The reader is referred to the review by Yubero, Montero, Santos-Ocaña, Salviati, Navas and Artuch [36], which outlines the studies to elucidate the genetic causes of CoQ10 deficiencies.

## 5. Supplementation with CoQ10

A number of disorders associated with both primary and secondary CoQ10 deficiency have been successfully treated via CoQ10 supplementation. An example of a primary CoQ10 deficiency successfully treated by CoQ10 supplementation is cerebellar ataxia, an autosomal recessive disorder typically manifesting in childhood or early adulthood [67]. Early identification of CoQ10 deficiency in patients with cerebellar ataxia is essential, since patients may show remarkable clinical improvement following CoQ10 supplementation when administered at an early stage of disease [67]. For example, clinical studies by Musumeci et al. [68] and Lamperti et al. [69] reported significant improvement in cerebellar function in children or young adults following CoQ10 supplementation (300–3000 mg/day). However, it should be noted that although cerebellar ataxia may be responsive to CoQ10 supplementation, the response to CoQ10 supplementation in clinical studies relating to other neurological disorders associated with a CoQ10 deficiency has in general been disappointing. This in turn may be related to the limited ability of CoQ10 to cross the blood–brain barrier [41,55]. In view of the limitations of absorption and bioavailability associated with CoQ10 therapy, the use of analogs of CoQ10 precursors has been investigated in an attempt to increase endogenous CoQ10 biosynthesis [70]. Pre-clinical studies have recently demonstrated the therapeutic potential of β-resorcylic acid (β-RA), a structural analog of the CoQ10 biosynthetic precursor 4-hydroxybenzoic acid, in the treatment of mitochondrial encephalopathy resulting from a CoQ10 deficiency. In view of the potential of β-RA to reduce the level of demethoxyubiquinone-10, the substrate of the COQ7 enzyme, it has been suggested that β-RA should be considered for the treatment of patients with CoQ10 deficiency as the result of mutations in either the *COQ4*, *COQ7* or *COQ9* genes [70]. However, following CoQ10 supplementation, clinical improvement following CoQ10 supplementation has been reported in patients with mutations in, *COQ2*, *COQ4*, *COQ6* and *ADCK3* genes [71,72].

An example of successful treatment of a secondary CoQ10 deficiency was the Q-SYMBIO clinical study of CoQ10 supplementation in patients with heart failure [73]. Patients with NYHA class III or IV heart failure were given supplemental CoQ10 (3 × 100 mg/day for two years), in addition to conventional heart failure medication. Supplementation with CoQ10 significantly reduced the relative risk of both cardiac related deaths (43%) and all-cause mortality (42%). There was no significant difference in adverse events between the CoQ10-treated and placebo groups over the duration of the study. Sub-group analysis of the effect of CoQ10 supplementation in the European cohort (231 of 420 patients) of the Q-SYMBIO study has now been undertaken [74]. In these patients, the relative risk of MACE (Major adverse cardiovascular events) was reduced by 67%, cardiac-related mortality by 53% and all-cause mortality by 55%; in addition, left ventricular ejection fraction was significantly improved by 6%, which was not observed in the original study. The improved outcome in the European cohort probably results from better patient compliance with supplementing CoQ10, resulting in a consistently higher plasma CoQ10 level for the duration of the study—3.4 mcg/mL at three months and 3.6 mcg/mL at two years, compared to 3.0 mcg/mL at three months and 2.1 mcg/mL at two years in the full cohort.

There is also evidence that supplementation with CoQ10 can benefit clinical status in patients with type II diabetes, chronic kidney disease and liver disease. Thus, several studies have reported significantly reduced blood CoQ10 levels in type II diabetic patients, correlating with increased levels of plasma glucose, HbA1C and markers of oxidative stress [75]. Kolahdouz et al. [76] reported that CoQ10 supplementation (200 mg/day for three months) significantly reduced HbA1c levels in type II diabetics. Similarly, Zahedi et al. [77] found that CoQ10 supplementation (150 mg/day for three months) significantly improved fasting plasma glucose and HbA1C levels, and Hosseinzadeh-Attar et al. [78] reported a significant improvement in HbA1c levels following supplementation with 200mg/day for three months. The benefit of CoQ10 supplementation on glycaemic control and blood lipid levels has been confirmed in a recent meta-analysis by Zhang et al. [79].

Similarly, plasma CoQ10 levels have been reported to be significantly lower in chronic kidney disease (CKD) patients (with or without haemodialysis), compared to healthy controls [80,81,82]. CoQ10 supplementation may improve renal function and reduce the need for dialysis in patients with CKD. In a randomised controlled study [83], 97 CKD patients were given supplementary CoQ10 (3 × 100 mg daily for three months) or a placebo. There was a significant improvement in markers of renal function (e.g., serum creatinine) in CoQ10-supplemented patients compared to the placebo, in both dialysed and non-dialysed patients. In particular, the number of patients requiring dialysis in the CoQ10-treated group decreased from 21 to 12, while remaining unchanged at 24 in the placebo group. Decreased CoQ10 levels may be a particular issue in CKD patients prescribed statins, since some studies have reported a deficit in CoQ10 status in association with this type of pharmacotherapy in a subset of patients. In addition to CKD, a manifestation of CoQ10 deficiency arguably more responsive to CoQ10 supplementation is nephrotic syndrome (as discussed in Section 3) [84].

With regard to liver disease, [85] found that blood CoQ10 levels were depleted in non-alcoholic fatty liver disease (NAFLD) patients, with the decrease in CoQ10 status correlating with increased liver inflammation and cirrhosis. A randomised controlled trial by Farhangi et al. [86] found that supplementation with CoQ10 (100 mg/day for four weeks) resulted in decreased systemic levels of biochemical markers of inflammation and oxidative stress. In the most recent randomised controlled trial [87], NAFLD patients given 100 mg CoQ10 per day for three months showed significant reductions in the levels of blood markers for liver inflammation and damage (aspartate aminotransferase, gamma-glutamyl peptidase, C-reactive protein).

As a result of the potential variability in the quality of CoQ10 supplements, it is important to note that any CoQ10 used in clinical studies should be manufactured to pharmaceutical standards and be of documented bioavailability in human subjects. When supplemental CoQ10 is first produced (via a yeast fermentation process), it is obtained in the form of crystals that cannot be absorbed from the digestive tract. It is essential that these crystals are dispersed into single CoQ10 molecules (and remain dispersed during the product shelf-life) to enable optimum bioavailability; the absence of such crystal dispersion in supplemental CoQ10 formulations reduces bioavailability in human subjects by 75% [88].

## 6. Conclusions

CoQ10 has a central role in the metabolism of all cells, and a CoQ10 deficiency is linked to the pathogenesis of a range of disorders. Primary CoQ10 deficiency results from genetic defects in the multi-step CoQ10 biosynthetic pathway. Brain, muscle and kidney tissues are particularly susceptible to the metabolic consequences of a deficit in the status of this isoprenoid, presenting clinically with disorders such as ataxia, myopathy and nephrotic syndrome, respectively. Early identification of such primary CoQ10 deficiencies is essential, since patients may show remarkable clinical improvement following CoQ10 supplementation when administered at an early stage of disease. Secondary CoQ10 deficiency has been identified in a wide range of disorders, including cardiovascular disease, chronic kidney disease, type II diabetes and liver disease; depletion of CoQ10 may result in part from the increased levels of OS in these disorders. Again, significant symptomatic improvement in these disorders, particularly in heart failure, has been reported following CoQ10 supplementation. Finally, it is important to emphasise that any CoQ10 supplements used in such clinical studies are produced to pharmaceutical standards, and that CoQ10 crystals in supplemental products are adequately dispersed to ensure optimal bioavailability.

## Abbreviations

CKD	Chronic kidney disease
CoQ10	Coenzyme Q10
METC	Mitochondrial electron transport chain
NAFLD	Non-alcoholic fatty liver disease
OS	Oxidative stress
